# Population-Based Screening of Newborns: Findings From the NBS Expansion Study (Part One)

**DOI:** 10.3389/fgene.2022.867337

**Published:** 2022-07-22

**Authors:** Amy Brower, Kee Chan, Marc Williams, Susan Berry, Robert Currier, Piero Rinaldo, Michele Caggana, Amy Gaviglio, William Wilcox, Robert Steiner, Ingrid A. Holm, Jennifer Taylor, Joseph J. Orsini, Luca Brunelli, Joanne Adelberg, Olaf Bodamer, Sarah Viall, Curt Scharfe, Melissa Wasserstein, Jin Y. Chen, Maria Escolar, Aaron Goldenberg, Kathryn Swoboda, Can Ficicioglu, Dieter Matern, Rachel Lee, Michael Watson

**Affiliations:** ^1^ American College of Medical Genetics and Genomics (ACMG), Bethesda, MD, United States; ^2^ Geisinger Health System, Danville, PA, United States; ^3^ Division of Genetics and Metabolism, Department of Pediatrics, University of Minnesota Twin Cities, Minneapolis, MN, United States; ^4^ School of Medicine, University of California, San Francisco, San Francisco, CA, United States; ^5^ Mayo College, Ajmer, India; ^6^ Wadsworth Center, New York State Department of Health, Albany, NY, United States; ^7^ Connectics Consulting, Atlanta, GA, United States; ^8^ Department of Human Genetics, Division of Medical Genetics, Emory University, Atlanta, GA, United States; ^9^ Department of Pediatrics, University of Wisconsin-Madison, Madison, WI, United States; ^10^ Boston Children’s Hospital, Harvard Medical School, Boston, MA, United States; ^11^ Division of Neonatology, The University of Utah, Salt Lake City, UT, United States; ^12^ MedStar Heart and Vascular Institute, Fairfax, VA, United States; ^13^ Departments of Molecular & Medical Genetics and Pediatrics, Oregon Health and Science University, Portland, OR, United States; ^14^ Department of Pediatrics, Yale University, New Haven, CT, United States; ^15^ Albert Einstein College of Medicine, New York, NY, United States; ^16^ Center for Genomic Medicine, Harvard University, Cambridge, MA, United States; ^17^ Department of Pediatrics, Children’s Hospital of Pittsburgh, Pittsburgh, PA, United States; ^18^ Department of Bioethics and Medical Humanities, Case Western Reserve University, Cleveland, OH, United States; ^19^ Massachusetts General Hospital Cancer Center, Boston, MA, United States; ^20^ Children’s Hospital of Philadelphia, Philadelphia, PA, United States; ^21^ Mayo Clinic, Rochester, MN, United States; ^22^ Texas Department of State Health Services, Austin, TX, United States; ^23^ Washington University School of Medicine (Adjunct), St. Louis, MO, United States

**Keywords:** research, genomics, ACMG, NBSTRN, newborn screening

## Abstract

Each year, through population-based newborn screening (NBS), 1 in 294 newborns is identified with a condition leading to early treatment and, in some cases, life-saving interventions. Rapid advancements in genomic technologies to screen, diagnose, and treat newborns promise to significantly expand the number of diseases and individuals impacted by NBS. However, expansion of NBS occurs slowly in the United States (US) and almost always occurs condition by condition and state by state with the goal of screening for all conditions on a federally recommended uniform panel. The Newborn Screening Translational Research Network (NBSTRN) conducted the NBS Expansion Study to describe current practices, identify expansion challenges, outline areas for improvement in NBS, and suggest how models could be used to evaluate changes and improvements. The NBS Expansion Study included a workshop of experts, a survey of clinicians, an analysis of data from online repositories of state NBS programs, reports and publications of completed pilots, federal committee reports, and proceedings, and the development of models to address the study findings. This manuscript (Part One) reports on the design, execution, and results of the NBS Expansion Study. The Study found that the capacity to expand NBS is variable across the US and that nationwide adoption of a new condition averages 9.5 years. Four factors that delay and/or complicate NBS expansion were identified. A companion paper (Part Two) presents a use case for each of the four factors and highlights how modeling could address these challenges to NBS expansion.

## 1 Introduction

Each year in the United States (US), at least 12,905 (Sontag et al., 2020) infants are identified with a genetic disease through the multi-component, multi-stakeholder system of newborn screening (NBS). NBS is recognized as one of the most successful public health programs in the US (Centers for Disease Control: Morbidity and Mortality Weekly Report (MMWR)) because it provides the opportunity to identify at-risk infants in a population regardless of race, income, or location of birth. Early identification of these at-risk infants facilitates timely diagnosis and administration of often life-saving treatment.

NBS began in the 1960s when a longitudinal study funded by the *Eunice Kennedy Shriver* National Institute of Child Health and Human Development (NICHD) discovered that newborns who were identified as having phenylketonuria (PKU) on a screening test using a blood spot on filter paper taken shortly after birth benefited from early diagnosis and treatment (Alexander, 2003). This discovery led to newborn screening pilots for PKU in several states and eventual nationwide screening of essentially all newborns using state-based public health laboratories.

Over the past 60 years, the number of possible screened conditions has increased from 1 to 81, with 75% (61/81) of these conditions recommended for screening by a federal advisory committee (Advisory Committee on Heritable Disorders in Newborns and Children (Advisory Committee on Heritable Disorders in Newborns and Children, 2010) Recommended Uniform Screening Panel (RUSP) (Advisory Committee on Heritable Disorders in Newborns and Children, 2011 RUSP)). Sixty-one conditions are included in the RUSP, and an additional 20 conditions are screened in at least one state as reported to the Association of Public Health Laboratories (Association of Public Health Laboratories Newborn Screening, 2020) Newborn Screening Technical assistance and Evaluation Program (NewSTEPs). This increase is largely due to advances in screening methodologie,s including the development of tandem mass spectrometry (MS/MS) in particular. The feasibility of screening for more than one condition using a single technology platform dramatically increased the number of conditions amenable to NBS (Ombrone et al., 2016; Farrell et al., 2020; Martin et al., 2020). In the future, the addition of genomic technologies to NBS would similarly increase the number of conditions that are candidates for NBS.

The composition of NBS panels and screening recommendations have been based on Wilson and Jungner’s criteria as outlined in “Principles and practice of mass screening for disease” (Wilson and Jungner, 1968). In addition, consideration for adding a condition to NBS panels has historically required onset in the neonatal period and effective treatment early in life thapreventsed or significantly reduces morbidity and mortality (Watson et al., 2006). Treatment regimens have now evolved to include gene therapy, stem cell transplant, cochlear implants, surgical repair of congenital heart defects, enzyme replacement, and genotype-specific therapies, (Puck, 2019; De Vivo et al., 2019; Dabbous et al., 2019), leading to many more conditions for which there may be early, effective treatment. Moreover, even the tenet of early treatment is being challenged by the expansions of NBS panels to include conditions with later childhood and adult-onset forms.

As outlined in the Newborn Screening Saves Lives Reauthorization Act of 2014 (NBSSLA) (Senate of the United States, 2019), three federal agencies each play a key role in advancing and maintaining NBS. 1) The NICHD is charged with supporting NBS research, including funding and administering the Newborn Screening Translational Research Network (Newborn Screening Translational Research Network, 2011), as well as investigator-driven NBS research to discover novel screening, diagnostic, and treatment technologies, nd NBS research and implementation pilots. 2) The Health Resources and Services Administration (HRSA) is tasked with ensuring the availability of services and providers to care offorBS-screened patients, administering the Advisory Committee on Heritable Disorders in Newborns and Children (Advisory Committee on Heritable Disorders in Newborns and Children, 2016a), funding NewSTEPs and supporting state adoption pilots. 3) The CDC operates the Newborn Screening Quality Assurance Program (NSQAP), a national program that provides training and assesses the performance of state laboratories conducting screening (Centers for Disease Control and Prevention, 2020 NSQAP).

**FIGURE 1:**
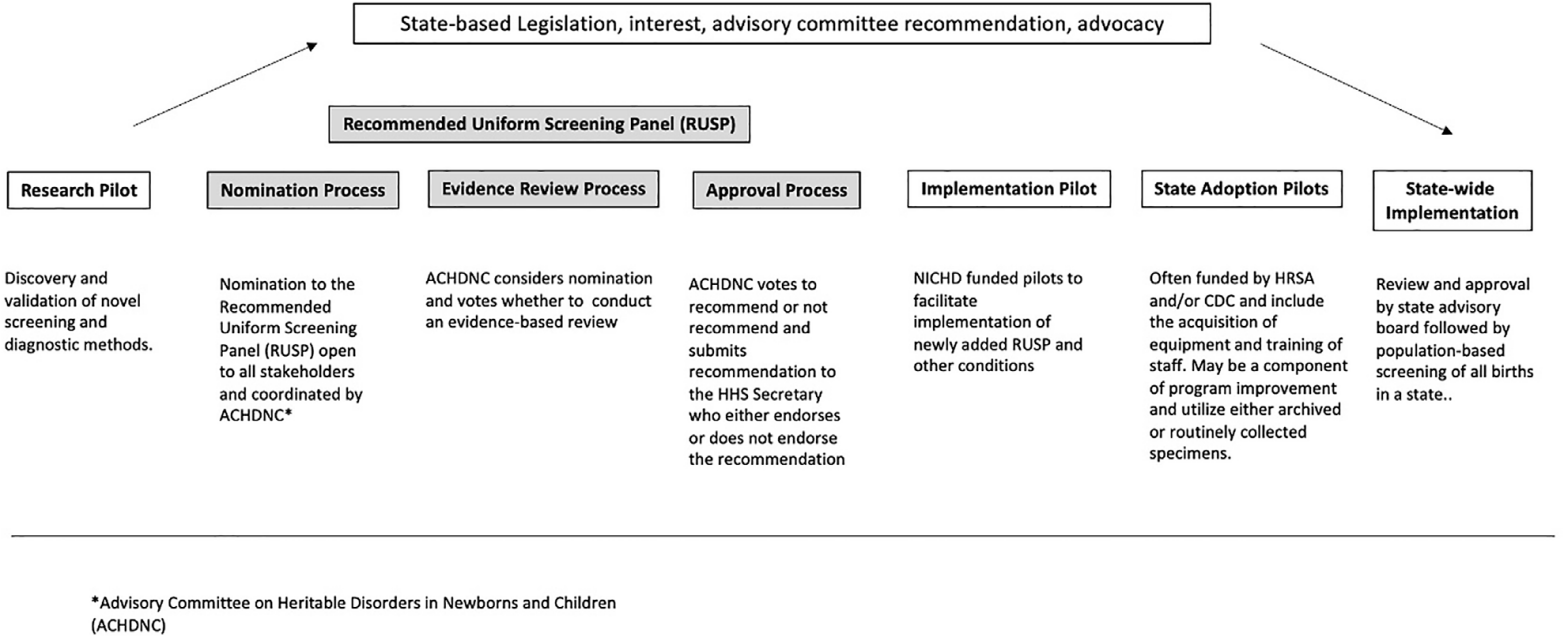
Pathway of candidate conditions.

Conditions are considered candidates for NBS based on the RUSP nomination criteria, which includes an assessment of whether early identification and intervention results in improved health outcomes. Figure 1 describes the different stages of NBS expansion from research pilots to nationwide implementation. As shown in the figure, an important step in understanding whether a condition is a candidate for NBS is to conduct research pilots of the entire screening process, including the screening test, diagnostic testing, clinical referral, and treatment, to assess the feasibility and potential benefits of early identification and intervention. Prospective or retrospective studies designed to assess the analytical and clinical validity of screening methods are often undertaken as an initial step. These studies or research pilots are typically a collaboration of multiple state NBS programs, working alone or with researchers, clinicians, and/or industry (diagnostics, medical device, and/or drugs), and they capture the initial performance of the screening test (Elliott et al., 2016). The second step is the implementation pilot. The NBSSLA authorized the Hunter Kelly Newborn Screening Research Program to conduct implementation pilot studies on conditions recommended by the (Advisory Committee on Heritable Disorders in Newborns and Children, 2016b) ACHDNC to ‘‘…ensure that screenings are ready for nationwide implementation.’’

In response, in 2016, NICHD created a pool of three states to conduct NBS pilots to facilitate the implementation of conditions recently recommended to the RUSP by the Health and Human Services (HHS) Secretary, and utilizing the coordinating infrastructure of the NBSTRN (Puryear et al., 2019). NBSTRN is a resource for investigators engaged in NBS-related research, led by the American College of Medical Genetics and Genomics (ACMG) and funded by a contract from NICHD. While these implementation pilots generate valuable information and data to accelerate and support the adoption of screening by state NBS programs, each state usually conducts state adoption pilots to demonstrate that they can meet the analytical standards established during the research and/or implementation pilots (Hall et al., 2020). Research pilots differ from implementation pilots, both of which differ from state adoption pilots (Vogel et al., 2015). However, all three types of pilots focus on the analytical validity of screening and diagnostic methods. All kinds of pilots are supported and funded through different mechanisms. Research pilots are supported by various stakeholders, including the Centers for Disease Control and Prevention (Centers for Disease Control and Prevention, 2011), the National Institutes of Health (NIH, usually NICHD), industry, and advocacy groups. Implementation pilots are funded by NICHD and utilize a task order for each pilot available to a pool of three states, currently New York, Georgia, and North Carolina. State adoption pilots are funded by the Health Resources and Services Administration (HRSA) and CDC and include the acquisition of equipment and training of staff. Enrollment in prospective research and implementation pilots occurs in birthing facilities and may require informed consent from parents. Prospective state adoption pilots are typically conducted as a component of program improvement and utilize either archived or routinely collected specimens.

Over the past decade, the NBSTRN coordinated pilot studies and worked with several researchers and disease advocacy organizations to compile data and review the scientific literature to facilitate the nomination of conditions to the ACHDNC in addition to the RUSP. To check current practices, identify expansion challenges, and propose strategies to evaluate changes and improvements to NBS expansion, NBSTRN designed and conducted the NBS Expansion Study. The study included an in-person workshop, a review of state Practices, completed Pilots, and efforts of the ACHDNC, and an expert opinion survey on the readiness of candidate conditions for NBS pilot studies.

## 2 Materials and Methods

### 2.1 NBS Expansion Study Workshop

Eighteen individuals participated in a 2 day workshop organized and hosted by NBSTRN staff. Attendees were selected based on their content knowledge of NBS, technology development and research, and involvement in NBS programs and pilots. Overviews of research, implementation, and state adoption pilots and expansion efforts were given by individuals from the NICHD pilot states, state NBS programs, and the HRSA-funded APHL NewSTEPs. The conference was recorded and transcribed. NBSTRN staff analyzed the transcripts and developed themes. These were presented to attendees for review, editing, and synthesis into a final report that was submitted to NICHD for consideration. The NBSTRN Steering Committee, a twelve-person group that guides NBSTRN activities, reviewed the workshop findings along with NICHD feedback and recommended NBSTRN survey state programs to assess their activities in the longitudinal follow-up of newborns confirmed with a diagnosis and conduct an expert opinion survey to compile and rank the growing pipeline of conditions that are candidates for NBS pilots and eventual RUSP nomination.

### 2.2 Review of NBS and Expansion Efforts to Date

The online resources, including the NewSTEPs and NBSTRN online repositories, were reviewed to gather information and data describing state screening panels and practices. State NBS program websites were searched to identify legislation that mandates screening for non-RUSP conditions and identify results of long-term follow-up of screen-positive cases. Publications and/or summary reports provided by pilot sites were reviewed to analyze the number of sites; screened, referred, and diagnosed newborns; and pilot duration for Severe Combined Immune Deficiency (SCID), Pompe Disease, Mucopolysaccharidosis Type I (MPS I), and/or X-Linked Adrenoleukodystrophy (X-ALD). The ACHDNC website, meeting transcripts, reports, and letters were reviewed to summarize information related to nominated conditions.

### 2.3 Expert Opinion Survey on Readiness of Candidate Conditions for NBS Pilot Studies

NBSTRN staff compiled a list of 46 candidate conditions and developed a questionnaire that provided the name of the condition, RUSP status (included, nominated), and listed informative biomarkers, analytical method, second-tier test, and available treatment(s). A five-point Likert scale ranging from “No” (1) to “Yes” (5) was used to rate each condition for three criteria that are key to an ACHDNC nomination: 1) Understanding of the Condition (severity/urgency); 2) Test Efficacy; and 3) Treatment Efficacy. Criteria could be ranked as “0’’ when the respondent had “no opinion” about the condition/criteria (Supplementary Tables S1, S2).

The first criteria relate to whether there is sufficient understanding of the condition in question. This is especially important because NBS expansion has revealed considerable clinical variability and incidence differences compared to predictions from the evidence review. With implementation, the clinical variability inherent in nearly all screened conditions becomes evident, uncovering in some cases variability that is striking.

The second criteria address the availability of a high-throughput, sensitive, and specific screening algorithm, including 1st and 2nd tier tests, performed either on dried blood spots (DBS) or *via* physiologic assessment at the bedside. While the ability of MS/MS to screen for multiple inborn errors of metabolism (IEMs) simultaneously on a single sample facilitated rapid NBS expansion since the early 2000s, it also further complicated screening because conditions that did not meet the evidence threshold for inclusion in NBS could be detected while screening for those conditions that did meet the evidence. Similarly, genome or exome sequencing has the potential to identify multiple disease-associated pathogenic gene variants in a single assay and redefines assessment from that of a single test for a single disease to the identification of numerous disease risks. Metabolomics, proteomics, and other-omics are expected to further complicate this assessment. The third concept relates to the availability of treatments and interventions. The modality, urgency, efficacy, effectiveness, and availability of proposed therapies are important components in considering a condition for NBS.

Subject matter experts from the NBSTRN expert workgroups who did not attend the workshop contributed to the survey’s design. The survey (Supplementary Table S1) administered *via* REDCap was distributed to 633 individuals, including 595 medical geneticists, metabolic disease experts, and laboratorians *via* the Society for Inherited Metabolic Disease (SIMD) email list and 38 NBSTRN users and researchers who conduct NBS pilots. The survey was open for 8 weeks, and two reminders to complete the survey were sent at weeks three and six. Likert scale responses were extracted from the survey, and the mean score for each criterion across respondents was computed. No opinion ratings were recorded as “0’’ and were excluded from mean score calculations. Based on consensus and review of the survey data from NBSTRN Steering Committee members, mean scores above or equal to 3.5, corresponding to the 70th percentile, were interpreted as a “yes” for the criteria (“Yes, this condition has a screening test,” “Yes this condition is severe/urgent,” “Yes this condition has a treatment”). Mean scores below the 70th percentile were interpreted as a “no” for the criteria (“No, this condition does not have a treatment”). Standard errors for each mean were calculated, and conditions were organized into groups based on the 70th percentile cut-off. A condition was deemed ready for pilot testing for NBS inclusion if the mean score for all three criteria (test, condition, and treatment) was ≥70%.

## 3 Results

The NBS Expansion Study utilized a workshop of NBS experts, a survey of clinicians, a literature review, and a review of online resources and key efforts (e.g., ACHDNC, HRSA, CDC, and NICHD activities) to understand NBS expansion in the US. The findings are summarized below and organized by topic and data source.

### 3.1 Literature, Online Resources, and Key Effort Review Findings

#### 3.1.1 NBS Expansion in the United States

In the US, 53 state-and territory-based programs conduct NBS. Before 2002, the number of screened conditions varied considerably from state to state ranging between 3 and 43 conditions. To address these differences, in 2002, the ACMG led a multi-year effort to survey experts and review the medical literature to assess the availability and characteristics of screening tests, the availability and complexity of diagnostic services, and the availability and efficacy of treatments for 84 conditions considered candidates for NBS. In 2005, this effort led to the original RUSP, with 29 core and 25 secondary conditions (Watson et al., 2006). After 3 years, all but one NBS program reported screening for all core conditions. This move to uniformity was achieved in a short timeframe because 80% (23/29) of the core conditions could be screened using a common multiplex technology, tandem mass spectrometry (MS/MS).

Using the ACMG effort as a model, the ACHDNC developed a nomination and evidence review system that is open to all stakeholders (Green et al., 2007). Since 2007, 13 conditions have been nominated and reviewed, and six were recommended for screening by the ACHDNC and, ultimately, the HHS secretary. Nomination to the RUSP is open to all stakeholders, and nominated conditions follow a standard process of consideration, including an evidence review. Conditions are usually reviewed one by one, and the review must be completed within 9 months. While the system in place to amend the RUSP encourages uniformity across the US by recommending conditions for screening, state programs are not obligated to follow those recommendations, and each state decides on the makeup of its state’s screening panel.

#### 3.1.2 Composition of NBS Panels

A review of current state panels in 2021 using online resources found that screening for up to 20 non-RUSP conditions is mandated legislatively in 23 states, representing deviations from the goal of the RUSP, which is uniformity based on evidence review (Table 1). These 23 states account for the screening of 54% (2,116,299/3,883,107) of US newborns (ACHDNC RUSP). Thirty-five percent (7/20) of the conditions have been nominated to the RUSP in the past (ACHDNC RUSP), and 8/20 (40%) are included in a completed or current pilot (ScreenPlus, 2022). Since our review, Mucopolysaccharidosis Type II (MPS II) was recommended to the RUSP in February 2022. The current status of state NBS panels can be found on the individual state websites, as well as the NewSTEPs Repository and the NBSTRN data tool called the NBS Conditions Resource (NBSTRN NBS-CR, 2022). The Newborn Screening Conditions Resource (NBS-CR) provides a centralized resource of facts and statistics on both screened and candidate conditions. The NBS-CR is designed to be an interactive resource for researchers, clinicians, parents, and families to learn more about these disorders and links to National Library of Medicine (NLM) resources, including the National Center for Biotechnology Information (NCBI). The NBS programs report several reasons for screening for conditions that are not on the RUSP, including state legislation, state advisory committee recommendation, and advocacy (The Brack Bills, 2016; Justia US Law, 2019; Connecticut General Assembly, 2020).

**TABLE 1:** NBS conditions screened in at least one state but not on RUSP.

Carbamoyl phosphate synthase (CPS) deficiency	Fabry disease	Hyperornithinemia with gyrate deficiency	Nonketotic hyperglycinemia
Congenital cytomegalovirus infection	Formiminoglutamic acidemia	Hyperornithinemia-hyperammonemiahomocitrullinemia syndrome	Ornithine transcarbamylase (OTC) deficiency
Congenital human immunodeficiency virus infection	GAMT deficiency	Krabbe Disease	Prolinemia Type I/Type II
Congenital toxoplasmosis infection	Gaucher disease	Mucopolysaccharidosis Type II	Pyroglutamic acidemia
Ethylmalonic encephalopathy	Glucose-6-phosphate dehydrogenase deficiency	Niemann Pick disease	Zellweger syndrome

#### 3.1.3 NBS Pilots

Pilots of conditions newly recommended to the RUSP are conducted in conjunction with at least one state-based NBS program to assess the analytical and clinical validity of the screening technology. A review of five of these pilots, shown in Table 2, found that the average duration of screening (which can include multiple state programs) was 8.8 months; the number of newborns screened ranged from 12,065 to 420,000; each pilot found at least one case; the screening technology and the follow-up algorithms used by each state varied; there was no coordination of data analysis or consensus developed by the participating states; not all states participated in every pilot; most pilot findings were either presented at scientific meetings or published within 3 years.

**TABLE 2:** NBS pilots after HHS endorsement for RUSP.

Condition	RUSP addition (month/Year)	Number of sites	Number of newborns screened	Screening start	Screening duration (months)	Number referred	Number diagnosed	Publication date (mont h/year)	Link to publication
SCID	2/2010	4^a^	167,509	10/2010	8	247	24	8/2014	https://pubmed.ncbi.nlm.nih.gov/32003821/
			420,000			43	1		
			32,000			8	7		
			34,544			9	4		
							8		
Pom pe	3/2015	2	59,332	1/2017	5.5	310	4	1/2020	https://pubmed.ncbi.nlm.nih.gov/32003821/
			108,862	NA		NA	13	NA^b^	NA
MPS I	2/2016	2	59,332	1/2017	5.5	17	11	1/2020	https://pubmed.ncbi.nlm.nih.gov/32003821/
			62,734	8/2016					
					7	1	1	8/2019	
						9	4		
X- AL D	2/2016	2	51,081	7/2017	5	12	4	NA	NA
			52,301	3/2018	4	1	8	1/2020	https://pubmed.ncbi.nlm.nih.gov/32003821/
						2			
SMA	7/2018	2	146,749	2/2019;	12	23	11	NA	NA
									https://www.ncbi.nlm.nih.gov/pmc/articles/PMC8006221/
			12,065	10/2018	15	2	1	3/2021	

a NY, CA, WI, conducted screening *via* courier for Louisiana; MA conducted screening *via* courier for Puerto Rico.

b NA, designates not published.

There are no standardized requirements or endpoints for NBS pilot studies, and the choice of outcomes and the development of robust statistical endpoints may be complicated because NBS conditions are rare and may have variable penetrance, age of onset, and severity. The endpoint for enrollment for some pilot studies is a defined period of time or population size. In contrast, others end once a single newborn with the targeted condition has been identified and the diagnosis confirmed. As a consequence of the design of both research and implementation pilots coupled with the rarity of most diseases, an assessment of the treatment and long-term health outcomes of NBS-identified individuals is not feasible. This makes it difficult to assess the utility of screening with regard to long-term outcomes, which has only occurred, at best, after population-based screening has been implemented. Additionally, there are currently no systematic approaches for assessing the ethical, social, or behavioral impact of screening for particular conditions on newborns and their families.

The collection of longitudinal health information from clinicians, educators, and others that care for these individuals is critical but very challenging given the variety of health care systems that hold relevant information on outcomes and the non-reimbursed effort currently required by care providers to enter follow-up data into systems created for long-term follow-up. Long-term follow-up is defined by each program based on state policies and legislation but usually involves collecting health information beyond diagnosis, treatment, and referral to clinical care. To make the collection of long-term follow-up data more streamlined, the NBSTRN developed the Longitudinal Pediatric Data Resource (LPDR), which includes common data elements (CDEs) developed by clinical experts and electronic case report forms for use by state NBS programs, researchers, and other stakeholders. NBSTRN aggregates the follow-up data in the LPDR and makes de-identified summaries publicly available (NBSTRN SCID).

#### 3.1.4 Length of Time to Implement a New RUSP Condition

A review of the implementation status from the NewSTEPs Data Repository (APHL NewSTEPs) found that the time to achieve screening across all 53 programs for the first condition added to the RUSP, SCID, was 10 years. This multi-year adoption process has been repeated for the other five conditions recommended to the RUSP, as shown in Table 3. The length of time for implementing a new condition led the ACHDNC to add an assessment of state readiness to the evidence review process. This assessment enables a better understanding of the capacity of states to expand screening and the resources required to support expansion. An assessment of the capacity of the health care system, including subspecialties, to confirm diagnoses in screen-positive infants and manage diagnosed infants would be informative and facilitate state adoption but is not part of the current process. As the number of conditions that would benefit from early identification and treatment through NBS increases, workshop participants noted that a failure to address resources for follow-up and long-term care would continue to negatively impact NBS as a system.

**TABLE 3:** Implementation status of new RUSP conditions (4/21).

Condition^a^	HHS recommendation to RUSP	Status	Years
SCID	2010	100% (53/53)	10
CCHD	2011	100% (53/53)	9
Pompe	2015	43% (23/53)	5+
MPS I	2016	39% (21/53)	4+
X-ALD	2016	34% (18/53)	4+
SMA	2018	43% (23/53)	2+

a SCID, Severe Combined Immunodeficiency; CCHD, Critical Congenital Heart Disease; MPS I, Mucopolysaccharidosis Type I; X-ALD, X-Linked Adrenoleukodystrophy; SMA, Spinal Muscular Atrophy.

### 3.2 Expert Opinion Survey

The survey was delivered electronically to 633 experts *via* the SIMD list serve, 55 logged into the survey, and 65% (36/55) completed the survey (Supplementary Table S1). Forty-six conditions were scored for three concepts, totaling 138 possible scores for each respondent. The number of respondents who ranked the three concepts for each condition varied because the survey allowed a response of “0’’ for “no opinion,” and this resulted in an average of 27 respondents per condition with a range of 13–36. Mean scores and standard errors were calculated for each concept and condition (Supplementary Table S2). Two conditions, congenital HIV and guanidinoacetate methyltransferase deficiency (GAMT), ranked above 80% (Likert rank 4) for all three criteria. An additional 13 conditions ranked equal to or above 70% (Likert ranked 3.5). Using 70% as a cut-off for each criterion resulted in 15 conditions ranked as ready for NBS pilots based on condition understanding, available test, and available treatment. Eight conditions lacked a therapy, an additional eight conditions lacked a screening test, 12 conditions had understanding but lacked treatment and test, and three conditions were ranked below the cut-off for all three criteria (Table 4).

**TABLE 4:** Conditions meeting the 70% threshold across concepts to identify readiness for NBS pilots.

Condition, test and treatment> 3.5(*n* = 15)	Condition and test> 3.5(*n* = 8)	Condition and treatment> 3.5(*n* = 8)	Condition > 3.5(*n* = 12)	All concepts <3.5(*n* = 3)
Acute neonatal bilirubin encephalopathy	Duchenne muscular dystrophy	BCKDK deficiency	Cerebrotendinous xanthomatosis	3-phosphoglycerate DH deficiency
AGAT deficiency	Fragile X	Brown vialetto van laere syndrome	Chr. 22 Deletion q11.2	Adenine phosphoribosyltransferase deficiency
Arginase deficiency	MPS IVA	CPS deficiency	Congenital toxoplasmosis	Pyruvate DH lipoic acid synthetase deficiency
Cbl C, D deficiency	MTHFR deficiency	Familial hypercholesterolemia	Creatine transporter deficiency	
Congenital HIV	NCL2 neuronal ceroid lipofuscinosis	NAGS deficiency	Cytomegalovirus
CPT1A Deficiency	Niemann Pick A/B disease	OTC Deficiency	Friedreich Ataxia
Fabry	MPS IIIA	Wilson Disease	Krabbe Disease
G6PD	Smith lemli opitz syndrome	Wolman Disease	Menkes Disease
GAMT deficiency			Metachromatic Leukodystrophy
Gaucher			Molybdenum cofactor Deficiency
Hemoglobin H disease			Niemann Pick C Disease
MPS II			Pyruvate carboxylase deficiency
MPS VI			
MPS VII
Pyridoxine responsive epilepsy

## 4 Discussion

The NBS Expansion Study explored the addition of conditions to nationwide NBS, surveyed experts to assess the readiness of conditions for NBS pilots, and described factors that delay and/or complicate expansion. Although the number of clinical experts who completed the survey was low, the individuals who completed the survey are involved in caring for newborns diagnosed with a condition through NBS. The pool of potential survey respondents was based on the SIMD list-serve, and the majority of these individuals may not be involved in NBS efforts. Future surveys of clinical experts may benefit from a targeted messaging campaign to encourage involvement.

The Study identified four factors that delay and/or complicate NBS expansion.

### 4.1 Variability in Screening Panels Persists

A review of individual state NBS screening panels found growing variation in state NBS panels and shows that the number of conditions screened ranges from a low of 32 core conditions to a high of 71 core, secondary, and non-RUSP conditions combined. A total of 81 different conditions are screened across the US. The makeup of screening panels is determined by each state’s NBS program, and each program develops its own screening and follow-up algorithms. Non-RUSP conditions are added to state NBS panels through the efforts of advocates and legislation. Over one-third of the non-RUSP conditions have been submitted for evidence review to the ACHDNC, and 40% are part of current or past pilots. Therefore, state panels may inform the content of future NBS expansions. Although the CDC and NewSTEPs organize training and funding to facilitate state adoption, there is no formal dissemination plan to share data from pilot studies; thus, the current pilot system fails to capitalize on opportunities to disseminate findings from individual state efforts.

### 4.2 The Short Duration of Pilots Limits Information About Interventions and Health Outcomes

While pilot sites are usually able to describe the diagnosis and initial disposition of the referred cases, the short duration of pilots often limits the description of health outcomes after treatment. This results in several missed opportunities, including the ability to: 1) advance understanding of the genetic disease; 2) connect the screening for a defined biomarker with improved outcomes; 3) identify gaps in evidence to be filled to support the nomination to the RUSP; 4) plan for the medical system impact of adding a condition to screening; and 5) document the effectiveness of early identification through NBS.

### 4.3 Recent RUSP Additions Expand the Definition of NBS

While NBS aims to identify infants with conditions that benefit from the intervention before the onset of symptoms and during the newborn period, recent RUSP additions have variable onset and/or defined late-onset forms manifesting far beyond the newborn period, if at all (e.g., Pompe, heterozygous X-ALD). A tool should be developed to assess the ethical, social, and behavioral impact of NBS for such disorders on newborns and families to identify and mitigate any potential harms to maximize the net benefit of screening prior to the addition of the condition to the RUSP.

### 4.4 The RUSP Nomination and Evidence Review Process has Capacity Constraints

The number of conditions candidates for NBS pilots and nationwide screening continues to increase. The approach of one-by-one nomination, review, implementation, and state adoption applied to this pipeline of candidates equates to decades of pilots designed to assess only the analytical part of the screening and the short-term follow-up aspect of a complex, multi-component system.

Although NBS has the potential to revolutionize genomic medicine through the population-based use of genomics to screen, diagnose and treat individuals with a genetic disease, current NBS expansion practices limit the realization of this promise. Findings from the NBS Expansion Study support the conclusion that the current approach to the expansion of NBS (i.e., one-by-one nomination, evidence review and HHS recommendation, implementation pilots, and state adoption) does not easily accommodate the hundreds of rare genetic disorders that could potentially benefit from NBS. The four factors identified in our study highlight weaknesses and gaps in the current system. Addressing these challenges will require innovative solutions so that the NBS system can be modernized and become responsive to the rapid advances in screening and diagnostic technologies, the emergence of novel therapies, and the expectations of the public (or families/advocates). Our companion paper, “Using Models to Address Challenges in Newborn Screening Expansion Study Part Two,” builds upon these findings, suggests and prioritizes solutions using some case studies and models, and outlines a potential future course for NBS in the US.

## Data Availability

The datasets presented in this study can be found in online repositories. The names of the repository/repositories and accession number(s) can be found in the article/Supplementary Material.
