# Supplementary material for: Population-Based Screening of Newborns: Findings From the NBS Expansion Study (Part One)
Source: Front Genet. 2022 Jul 22;13:867337. doi: 10.3389/fgene.2022.867337 (PMC9354846; doi:10.3389/fgene.2022.867337)
Supplement: Supplementary file 2 [file Table1.DOCX]

**Expert Opinion Survey**

| **BACKGROUND** |
| --- |
| Newborn screening (NBS) is recognized as one of the most successful public health programs in the United States because it provides the opportunity to identify infants in a population who are at-risk for a screened disease that can be treated, regardless of race, income, or location of birth. The pipeline of conditions that are candidates for NBS based on emerging therapies and improved testing is growing rapidly while the definition of the condition to which screening is targeted is evolving. |
| An important step in understanding whether a candidate condition is a fit for nationwide NBS is to conduct a pilot of the screening technology followed by diagnostic workup, treatment and long-term follow-up. Because there is increasing concern that capacity for the needed population-level pilot studies is limited, we are interested in better defining where tests/conditions are in the pipeline en route to NBS. |
| We have compiled a list of candidate conditions for NBS and invite your feedback on the appropriateness of each candidate for a NBS pilot study. The three primary parameters that are assessed are how well the condition is understood, is there a test that meets the requirements of use in NBS, and is there an effective treatment. We have included in the survey the analytes of treatments to be considered. We are interested in your views on how the conditions in the attached list meet these criteria, realizing that some questions can only be answered in a large general population level pilot study. In responding, please don’t overly lower your scores due to gaps that require unbiased general population data to answer. |
|  |
| **DIRECTIONS** |
|  |
| On the **Conditions Worksheet**, please rate the following on a scale of 1 (No) to 5 (Yes), where 0 indicates No Opinion: |
| 1. Understanding of the Condition |
| 2. Test Efficacy |
| 3. Treatment Efficacy |
|  |
| On the **Demographics Worksheet**, please provide your contact information so we can follow-up with any additional questions and provide you feedback on the overall results. We will not publish or share your individual responses and you are not required to provide this information to participate. |

**Demographics Worksheet**

| Name | FirstName LastName |
| --- | --- |
| Degree | B.A.; B.S.; B.S.N.; NP; M.A; M.P.H; M.S.; M.S.N.; APRN; J.D.; M.D.; Ph.D.; Pharm.D.; Psy.D.; Sc.D.; Other |
| Institution/Company/Affiliation | ABC Corporation |
| Primary Email | [name@place.org](mailto:name@place.org) |
| Board Certification | ABMGG; FACMG; ABGC CGC; Other |
| Subject Matter Expertise Area(s) | Biochemical Genetics; Clinical Genetics; Clinical Cytogenetics; Molecular Genetic Pathology; Dermatology; Immunology; Neurology; Obstetrics and Gynecology; Pathology; Pediatrics; Psychiatry; Urology; Other |

**Survey**

|  |  |  |  |  | **APPROPRIATENESS FOR NBS PILOT STUDY** | | |
| --- | --- | --- | --- | --- | --- | --- | --- |
| **CONDITION** * Already in RUSP ** Already nominated for addition to RUSP | **INFORMATIVE MARKERS in DBS (High ­ Low ¯)** | **ANALYTICAL METHOD in DBS** | **2^nd^ TIER TEST(S) in DBS N/A, not available  (High ­ Low ¯)** | **AVAILABLE TREATMENT** (diet, drugs, procedures) | **UNDERSTANDING OF CONDITION** (Severity/Urgency) 0=no opinion (No) 1 - 5 (Yes) | **TEST EFFICACY** 0=no opinion (No) 1 - 5 (Yes) | **TREATMENT EFFICACY** 0=no opinion (No) 1 - 5 (Yes) |
| OTC deficiency | Cit ¯ | MS/MS | NA | Diet Conjugating agent |  |  |  |
| CPS deficiency | Cit ¯ | MS/MS | NA | Diet Conjugating agent |  |  |  |
| NAGS deficiency | Cit ¯ | MS/MS | NA | Diet Conjugating agent |  |  |  |
| MTHFR deficiency (plus Cbl G, Cbl E) | Met ¯ | MS/MS | Hcy ­ | Betaine Other |  |  |  |
| Cbl C,D deficiency* | C3 ­, Met ¯ | MS/MS | MMA ­, Hcy ­ | Vit. B12 Carnitine |  |  |  |
| Arginase deficiency* | Arg ­ | MS/MS | N/A | Diet |  |  |  |
| BCKDK deficiency | BCAA ¯ (Val, Xle) | MS/MS | BCAA ¯ by LC-MS/MS | Diet |  |  |  |
| Pyruvate DH lipoic acid synthetase (HGCLAS) def. | Gly ­ BCAA ¯ (Val, Xle) | MS/MS | BCAA ¯ by LC-MS/MS | lipoic acid supplement not successful |  |  |  |
| Pyruvate carboxylase (PC) deficiency | Cit ­, Glu ¯ | MS/MS | N/A | Proposed treatment: thiamine, lipoic acid, AA supplement with asp asn glu gln |  |  |  |
| CPT1A deficiency* | C0 ­ | MS/MS | N/A | Fasting avoidance |  |  |  |
| Brown Vialetto Van Laere sdr. (riboflavin transporter) | Multiple ACs ­ | MS/MS | N/A | high dose riboflavin |  |  |  |
| GAMT deficiency ** | Guac ­ | MS/MS | Guac ­ by LC-MS/MS | Creatine |  |  |  |
| AGAT deficiency | Guac ¯ | MS/MS | Guac ¯ by LC-MS/MS | Creatine |  |  |  |
| Creatine transporter (CRTR) deficiency | Cre/Crn ­ | MS/MS | NA | Creatine, AA supplement with arg gly |  |  |  |
| 3-phosphoglycerate DH deficiency (PHGDH) | Ser ¯, Gly ¯ | MS/MS | NA | Serine |  |  |  |
| Fabry disease | GLA activity ¯ | DMF, MS/MS | NA | ERT |  |  |  |
| Gaucher disease | GBA activity ¯ | DMF, MS/MS | GPsy ­ | ERT, BMT |  |  |  |
| Krabbe disease | GALC activity ¯ | MS/MS | Psy ­, 30kb del | HSCT |  |  |  |
| Niemann Pick A/B disease | ASM activity ¯ | MS/MS | OxySterols ­ | BMT |  |  |  |
| Niemann Pick C disease | OxySterols ­ | MS/MS | OxySterols ­ | miglustat |  |  |  |
| Wolman disease (acid lipase def.) | Cholesterol ­, triglycerides | MS/MS, Other | N/A | Sebelipase alpha |  |  |  |
| Hunter syndrome (MPS II) | I2S activity ¯ | DMF, MS/MS | HS ­, DS ­ | ERT |  |  |  |
| San Filippo disease (MPS IIIA) | SGSH activity ¯ | Immunocapture, MS/MS | DS ­ | no current treatment |  |  |  |
| Morquio disease (MPS IVA) | GALNS activity ¯ | MS/MS, Immunocapture | KS ­ | ERT |  |  |  |
| Maroteaux-Lamy sdr (MPS VI) | ARSB activity ¯ | MS/MS, Immunocapture | DS ­ | ERT |  |  |  |
| Sly syndrome (MPS-VII) | GUSB activity ¯ | MS/MS | DS ­, C6S ­ | ERT |  |  |  |
| Metachromatic Leukodystrophy | ARSA activity ¯ | Immunocapture, MS/MS | NA | BMT |  |  |  |
| Familial hypercholesterolemia | Cholesterol ­ | MS/MS, Other | Genetic test; APOB, LDLR, PCSK9 | Statins |  |  |  |
| Smith Lemli Opitz syndrome | 7DHC ­, Cholesterol ¯ | MS/MS | NA | cholesterol, simvastatin |  |  |  |
| APRT deficiency | Adenine ­ | MS/MS | NA | Allopurinol, Febuxostat |  |  |  |
| Cerebrotendinous xanthomatosis (CTX) | Bile acids ­ | FIA-MS/MS | Bile acids by LC-MS/MS | Drugs (CDCA) |  |  |  |
| Chr. 22 deletion q11.2 | TRECs | PCR | NA | supportive (heart, calcium, infections, early intervention) |  |  |  |
| Menkes disease  (copper transport) | Copper, ceruloplasmin | Immunocapture, PCR | NA | Copper |  |  |  |
| Duchenne/Becker muscular dystrophy (DMD/BMD) | CK ­, Cre/Crn ­ | Immunofluorometry, MS/MS | *DMD* sequencing | Steroids, Stop codon read-throughs |  |  |  |
| Fragile X | triplet repeats ­ | DNA | NA | Early intervention |  |  |  |
| Friedreich Ataxia (FRDA) | FXN ¯ | Immunocapture | NA | Proposed treatment: Ibedenone |  |  |  |
| G6PD deficiency | G6PD | Fluorescence | NA | appropriate avoidance |  |  |  |
| Wilson disease | Ceruloplasmin ¯ | Immunocapture | NA | chelation therapy |  |  |  |
| Molybdenum cofactor def. | uric acid ↓ | MS/MS | NA | cPMP |  |  |  |
| Acute neonatal bilirubin encephalopathy | bilirubin ­ | Transcutaneous, bilirubinometer | NA | phototherapy |  |  |  |
| Pyridoxine responsive epilepsy | NGS | Sequencing | Pathogenic variant in ALDH7A1 | Pyridoxine (B6) |  |  |  |
| NCL2 neuronal ceroid lipofuscinosis | PPT1 activity ¯ | MS/MS | NA | cerliponase alfa (Brineura) for neuronal ceroid lipofuscinosis 2 |  |  |  |
| Hemoglobin H disease | Hb H | HPLC | NA | transfusion |  |  |  |
| Congenital HIV | viral RNA | PCR | NA | Antivirals |  |  |  |
| Congenital toxoplasmosis | IgG Ab | ELISA | NA | pyrimethamine, sulfadiazine, and leucovorin. |  |  |  |
| Cytomegalovirus (CMV) | Hearing | Audiometry | PCR | antivirals |  |  |  |
